# Does severity of motor impairment affect reactive adaptation and fall-risk in chronic stroke survivors?

**DOI:** 10.1186/s12984-019-0510-3

**Published:** 2019-03-22

**Authors:** Tanvi Bhatt, Shamali Dusane, Prakruti Patel

**Affiliations:** Department of Physical Therapy, College of Applied Health Sciences, University of Illinois at Chicago, 1919, W Taylor St, (M/C 898), Chicago, IL 60612 USA

**Keywords:** Perturbation training, Adaptation, Motor impairment, Stroke

## Abstract

**Background:**

A single-session of slip-perturbation training has shown to induce long-term fall risk reduction in older adults. Considering the spectrum of motor impairments and deficits in reactive balance after a cortical stroke, we aimed to determine if chronic stroke survivors could acquire and retain reactive adaptations to large slip-like perturbations and if these adaptations were dependent on severity of motor impairment.

**Methods:**

Twenty-six chronic stroke participants were categorized into high and low-functioning groups based on their Chedoke-McMaster-Assessment scores. All participants received a pre-training, slip-like stance perturbation at level-III (highest intensity/acceleration) followed by 11 perturbations at a lower intensity (level-II). If in early phase, participants experienced > 3/5 falls, they were trained at a still lower intensity (level-I). Post-training, immediate scaling and short-term retention at 3 weeks post-training was examined. Perturbation outcome and post-slip center-of-mass (COM) stability was analyzed.

**Results:**

On the pre-training trial, 60% of high and 100% of low-functioning participants fell. High-functioning group tolerated and adapted at training-intensity level-II but low-functioning group were trained at level-I (all had > 3 falls on level-II). At respective training intensities, both groups significantly lowered fall incidence from 1st through 11th trials, with improved post-slip stability and anterior shift in COM position, resulting from increased compensatory step length. Both groups demonstrated immediate scaling and short-term retention of the acquired stability control.

**Conclusion:**

Chronic stroke survivors are able to acquire and retain adaptive reactive balance skills to reduce fall risk. Although similar adaptation was demonstrated by both groups, the low-functioning group might require greater dosage with gradual increment in training intensity.

## Introduction

Approximately more than 800,000 individuals annually suffer from stroke and its associated detrimental long term disability in the USA [1]. The primary deficits associated with stroke, such as sensorimotor impairment, postural dysfunction, and cognitive impairment, result in secondary complications such as falls [2–4]. The high risk of falls during the acute phase persists even into the chronic phase when people with chronic stroke (PwCS) regain their ambulatory ability; especially predisposing them to falls from unexpected environmental perturbations such as slips or trips [5].

Reactive balance control plays a crucial role in recovering from large unexpected perturbations, thereby lowering fall-risk [6–10]. A rapid and sufficiently large stepping response helps to regain postural stability by restoring the displaced base of support (BOS) and providing the necessary lever arm to generate adequate rotational counter-torque at step touchdown to decelerate the body’s forward or backward moving center of mass (COM) [11]. Studies examining reactive responses to stance perturbations in PwCS have reported delayed onset latencies of lower extremity muscles, with smaller amplitude and altered sequence of activation [12, 13]. Moreover, PwCS often show delayed compensatory step initiation with a short compensatory step, or they exhibit an aborted step or multiple stepping responses; all of which compromise postural stability and increase fall-risk [14–20].

Considering the above-mentioned evidence, training reactive responses in PwCS could be critical for fall-risk reduction in this population. Leveraging the principle of task specificity, perturbation training elicits reactive motor adaptations and induces learning of effective recovery strategies by improving COM state stability control and compensatory stepping responses throughout trials [21, 22]. Even a single session of perturbation training has shown to induce longer-term reduction in laboratory-induced and real-life falls in healthy older adults [23]. However, limited evidence exists on the effect of perturbation training on acquiring adaptations for fall-risk reduction in PwCS.

The role of neuroplasticity, specifically reorganization of the sensorimotor cortex, in optimizing motor recovery and function for both skilled volitional and locomotor tasks in PwCS is well known [24–26]. It is also established that the cerebellum and cerebral cortex play a crucial role in acquiring locomotor-balance adaptations [27, 28]. While cerebellar stroke can impair acquisition of motor adaptation, this ability has been shown to be intact in cortical stroke [27, 29, 30]. Nonetheless, little is known whether adaptations within the reactive balance control system are possible post-stroke. A preliminary training study employing therapist-induced, small magnitude external pull-push perturbations showed reduction in daily falls for sub-acute stroke patients [31]. Such low intensity perturbations, while appropriate for individuals in the early phases of recovery, might not be challenging enough to mimic real-life perturbations faced by community-dwelling PwCS.

Previously, it has been established that there is a need for different dosage considerations when training patients with varying degrees of impairment in order to improve locomotor balance control [32]. However, there is lack of evidence on recommendations for an optimal perturbation training intensity for PwCS with different severity of motor impairment to suitably match their motor capabilities and ultimately induce reactive adaptation. It is also unknown if PwCS with varying levels of motor impairments could safely tolerate the perturbation intensity dosages provided to healthy young and older adults for training.

This study aimed to examine if PwCS could acquire reactive adaptation to large slip-like stance perturbations, and if adaptive gains differed based on the perturbation intensity and severity of motor impairment. We also examined if the adaptive gains could be scaled when exposed to a higher perturbation intensity and then retained over several weeks.

## Methods

### Participants

Twenty six community-dwelling people with self-reported diagnosis of chronic (> 6 months) hemi-paretic, cortical stroke, confirmed by their physician, who were able to ambulate independently with or without an assistive device were included in the study. Participants were screened and excluded if they had cognitive impairment (≤ 26/30 on Montreal Cognitive Assessment Scale), aphasia (≥ 71/100 on Mississippi Aphasia Screening Test), low bone density (T score < − 1.5 on heel ultrasound), or presence of any other self-reported neurological, musculoskeletal, or cardiovascular condition. Clinical measures such as Berg balance test (BBS), Timed up and go test (TUG), 6 min walk test, 10 m walk test, and Chedoke-McMaster Stroke Assessment (CMSA) were performed to determine the baseline functional status and the severity of impairment. The demographic details of the participants are presented in Table 1. The study was approved by the institutional review board of the University of Illinois at Chicago.

**Table 1:** Demographics and clinical outcome measures of study participants

	High functioning group	Low functioning group
	Mean (SD); *n* = 15	Mean (SD); *n* = 11
Age (years)	58 (9.48)	59.54 (6.67)
Chronicity (years)	7.87 (5.4)	9.34 (4.2)
Impairment level		
CMSA (Leg)	4.93 (1.12)	4 (0.96)
CMSA (Foot)	3.86 (2.15)	2.09 (0.51)
Stroke type (hemorrhagic/ischemic)	7/8	3/8
Balance (BBS)	46.06 (6.22)	40.72 (7.01)
TUG (s)	15.19 (3.88)	17.48 (9.91)

### Training and testing protocol

**Fig. 1:**
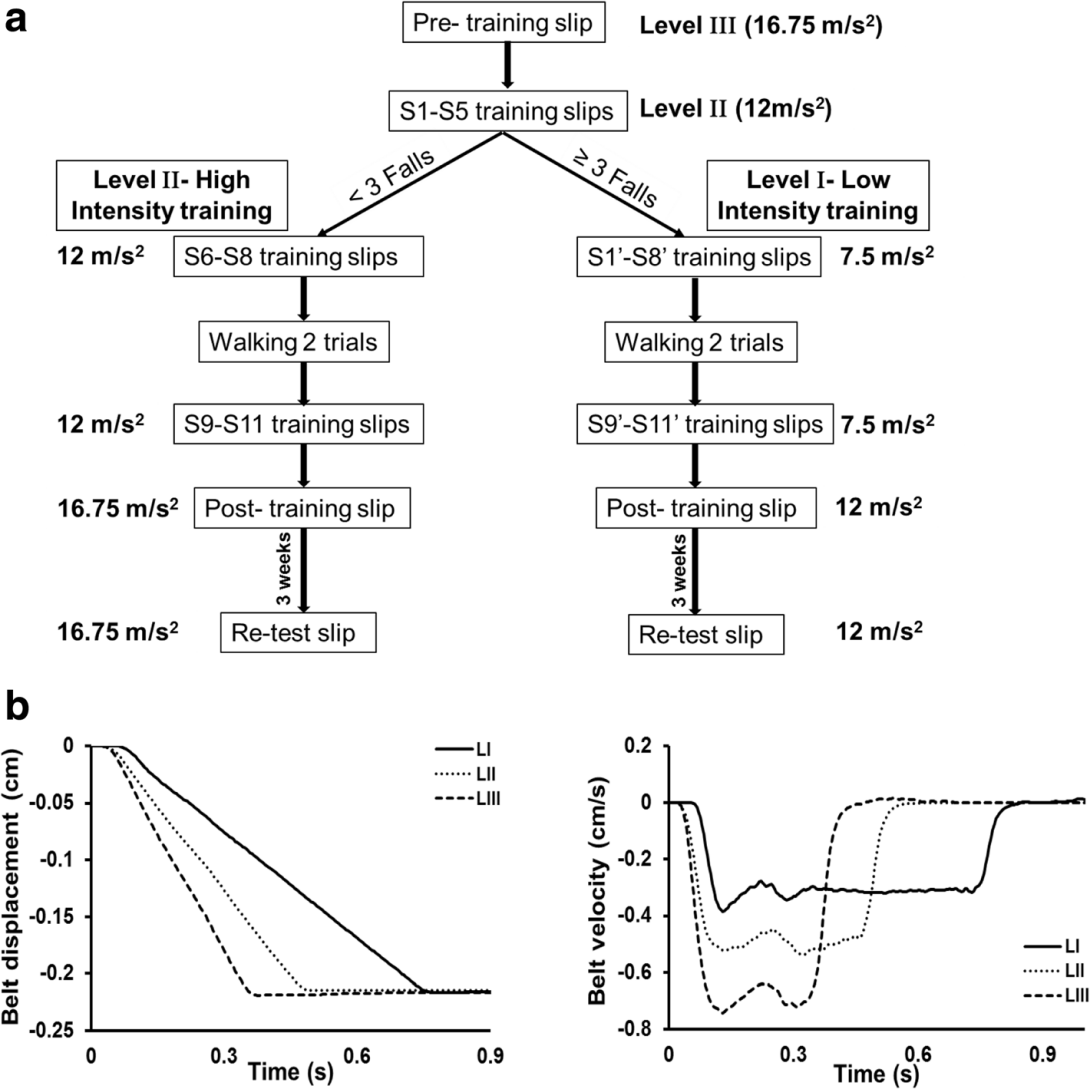
**a** Shows the research design and experimental protocol along with the intensities of perturbation for pre-training, training, post-training, and retest trials. The mark ‘indicating perturbation trials at lower intensity. **b** Shows the trajectory of belt displacement and the velocity profiles for the different perturbation intensities (level I, II and III) used for assessment and training

Participants were divided into two groups, a high functioning and a low functioning group, depending on their level of impairment as assessed by the Chedoke-McMaster Stroke Assessment scale (CMSA). Based on the findings from a previous study demonstrating differences in CMSA leg and foot scores between individuals with failed and successful reactive stepping response, the leg score was used to divide participants into two groups (> 4 designated as high functioning and ≤ 4 as low functioning) [33]. Participants were instructed to assume a standing position with feet shoulder width apart on the Activestep treadmill (Simbex, Lebenon, NH) which would deliver the slip-like, forward perturbation. All participants donned a safety harness suspended from the treadmill arch in order to prevent a fall where their knees would touch the treadmill belt. They were instructed to expect an unannounced slip-like perturbation at any instance. Participants were asked to perform their natural response to recover from the perturbation and prevent themselves from falling. After familiarization trials, participants were subjected to a pre-training trial at the highest intensity (level III), followed by five perturbation trials given at one lower intensity (level II). During these trials, if participants experienced more than three falls, they were assigned to be trained at a lower perturbation intensity (level I), whereas the rest of the participants continued to receive the remaining training at level II to complete the protocol consisting of a total of 11 slip trials. Thus, those participants who did not tolerate the higher intensity were exposed to 11 more slips at a lower intensity (S1’-S8’; S9’-11′) whereas, for the participants who tolerated the high intensity, these 5 trials were considered a part of the high intensity training arm and they received 6 (S6-S8; S9-S11) additional trials at this intensity (Fig. 1a).

Following the initial eight slip trials, all the participants were made to walk at their self-selected natural speed for two trials which served as wash-out trials in order to reduce anticipation of the upcoming perturbation. After training, both groups were subjected to a post-training trial at the pre-training intensity (level III for high intensity training arm and level II for low intensity training arm) to examine scaling of the recovery response. Three weeks after training, participants from both groups were re-tested in order to assess the training retention. At this re-test, each participant received a single slip perturbation at the pre-training perturbation intensity (Fig. 1a). During the training and testing sessions, participants were intermittently asked if they experienced any pain, discomfort, fatigue or fear and would like to discontinue. All the participants consented to complete the protocol.

### Data collection

An eight camera motion capture system (Motion Analysis Corporation) with a sampling rate of 120 Hz was used to record full body kinematics. Helen Hayes marker set with 29 markers positioned on bilateral bony landmarks, head and trunk was used to compute the center of mass (COM) [34, 35]. An additional marker placed on the treadmill belt was used to detect perturbation onset. Data from the passive reflective markers underwent low pass filtering using the fourth order Butterworth filter with a cut off frequency of 6 Hz. A load cell connected in series with the harness recorded the amount of body weight exerted on the harness during each trial and was synchronized with the motion capture system through an analog to digital convertor. Kinematic variables were calculated by using custom written algorithms in MATLAB (MathWorks Inc).

#### Perturbation outcome

Following a perturbation-induced backward balance loss, the outcome of each slip-like perturbation trial was identified as a fall or recovery. A fall outcome was identified if the harness supported more than 30% of the participant’s total body weight over a 1 s period post-perturbation and/or if one or more researchers had to provide more than moderate assistance to support the participant in resuming the starting position. The fall and recovery outcomes were verified by visual inspection of video recordings [36–38]. Further, both outcomes could be associated with a compensatory strategy in the form of a backward step or an aborted step, or a no step response. The backward stepping response was identified when there was clearance of both the heel and toe from the treadmill surface and the stepping limb landed posterior to the non-stepping limb. An aborted step occurred when individuals unloaded one of their heels in order to initiate a step followed immediately by reloading of the foot without complete toe clearance [19, 39, 40]. A no step response occurred when either the participant recovered from the balance loss without stepping or fell in the harness without even an attempt to initiate a stepping response. Unloading of the foot was determined from the Z-coordinate of the stepping limb heel marker. The number of compensatory steps executed to recover balance were also recorded.

#### Center of mass state stability

The COM position was measured relative to the most posterior margin of the BOS (i.e. the heel of the stepping limb at step touchdown) and was further normalized by the participants’ foot length. The COM velocity was derived from the first order differentiation of the COM position and was also expressed relative to the heel velocity of the stepping limb at step touchdown. It was normalized by a dimensionless fraction of √g*h where *g* is the acceleration due to gravity and *h* is the body height in meters [41]. The COM state stability at touchdown was measured as the shortest distance of the instantaneous COM state from the theoretical boundary established for backward loss of balance [42–44]. Stability values < 0 indicate that the COM state lies below the theoretical backward balance loss threshold and implies instability in the backward direction while values > 0 indicate a more stable COM state.

#### Other kinematic variables

Compensatory step length was recorded as the antero- posterior displacement of the stepping limb heel post-perturbation from liftoff to its touchdown and was normalized to the participant’s body height. Trunk angle in the sagittal plane was computed by subtracting the absolute peak trunk angle (highest value between lift off to touchdown) from the trunk angle at perturbation onset. Positive peak trunk angle values denoted trunk flexion and negative values denoted trunk extension relative to the vertical axis.

### Statistical analysis

All the participants in the low functioning group (based on their CMSA scores) were unable to tolerate training at level II, so this group will be referred as the LFLI (low functioning, low intensity group) throughout the text. All the participants designated to the high functioning group, however, were able to tolerate training at level II and will, therefore, be referred as the HFHI (high functioning, high intensity group). A Generalized estimating equations (GEE) model was used to determine changes in the binary outcome (fall incidence) between groups and across trials. Overall significant effects were resolved by conducting GEE for each group. Significant effects were further analyzed with planned comparisons using McNemar’s test to compare differences between specific trials (between pre-training and T1; T1 and T5; pre-training and T5; T6 and T11). The model was processed separately for early (pre-training, T1, and T5) and late (T6, T11) adaptation phases. Similarly, a mixed effect model analysis was conducted separately for early and late adaptation phases to determine changes in non-parametric (number of compensatory steps) and parametric (stability) measures between groups and across trials. Significant trial and group effects were then analyzed with planned comparisons using paired and unpaired t-tests for the stability measure and with Wilcoxon and Mann Whitney U tests for number of compensatory steps. To further examine the relationship between variables, a bivariate Pearson correlation analysis was performed between COM position and velocity with stability, with pooled data from both groups. A similar relationship was examined between step length and trunk angle with COM position.

For analyzing the scaling and retention effect, similar GEE and mixed effects models were used to compare fall incidence (GEE), number of compensatory steps and stability (mixed models) between the pre-training, post-training, and retest trials. Significant effects were analyzed by planned comparisons using previously mentioned tests. All analyses were performed using SAS 9.4 (Cary, NC) and SPSS version 24 with a significance level of 0.05.

## Results

### Trial to trial adaptation

#### Early adaptation

**Fig. 2:**
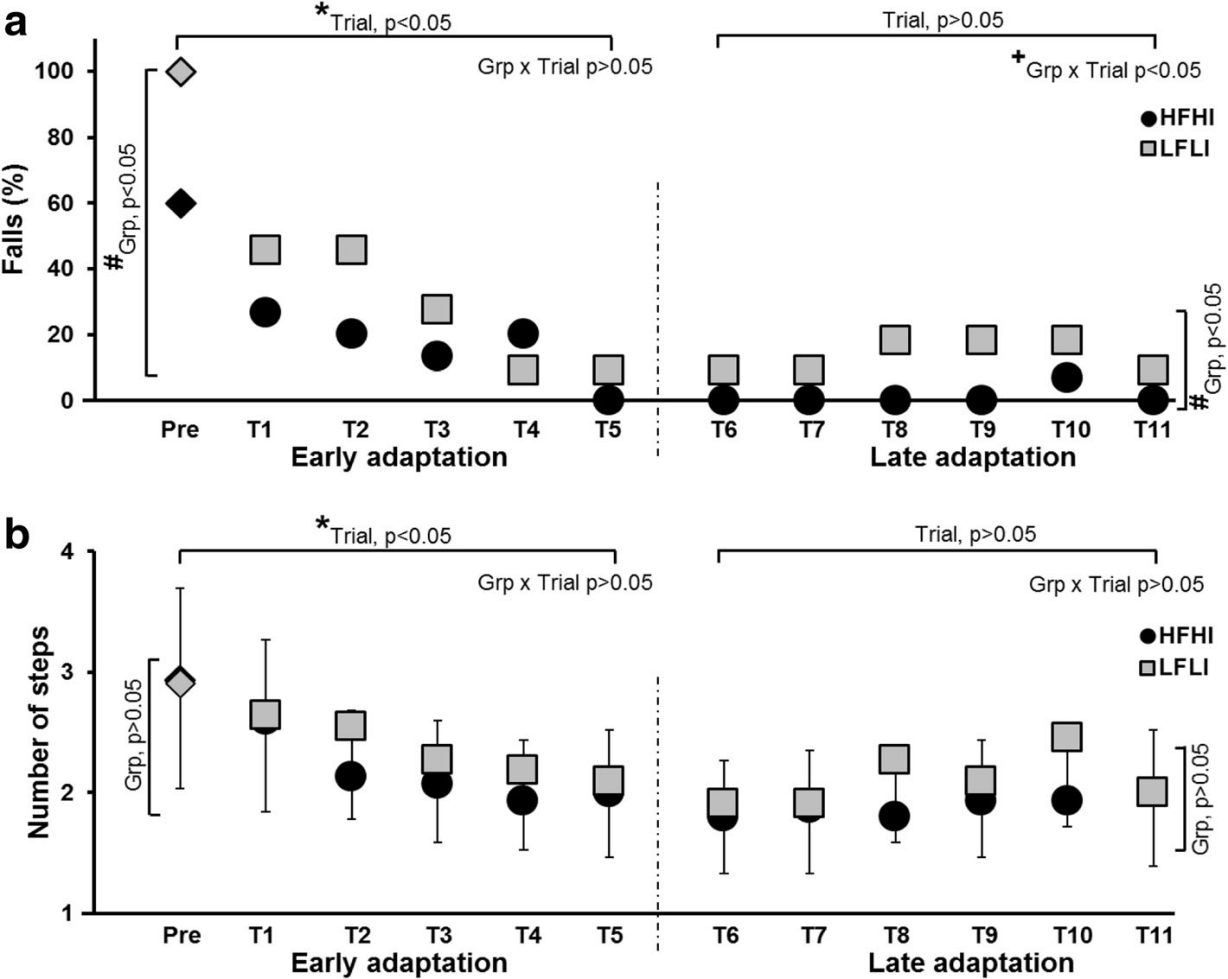
**a** Demonstrates the change in fall percentage during the pre-training and training trials for both the HFHI and LFLI groups. The LFLI group when subjected to five slip perturbations at one intensity (level II) lower than pre-training trial (level III) experienced falls on all five trials, which is not shown in the figure. **b** Demonstrates early and late adaptive changes in the number of compensatory steps taken after slip perturbation. *, indicates a significant main effect of trial, # indicates a significant main effect of group and + indicates significant group x trial interaction.

All the participants experienced a backward loss of balance after being exposed to level III pre-training perturbation, demonstrating an initial backward compensatory stepping response (22/26) or an aborted step (4/26) followed by multiple stepping, with or without a fall (Fig. 2a). Following the pre-training trial, both groups at their respective training intensities exhibited reduced fall incidence and improved compensatory stepping response.

The GEE model demonstrated a significant group effect (*p* < 0.05) and trial effect (*p* < 0.001) for fall percentage in the early phase (Fig. 2a) with no group*trial interaction (*p* > 0.05). Both groups exhibited improvement in fall incidence during the early trials, with fall reduction from 100% at pre-training to 45% at T1 and 9% at T5 for the LFLI group (trial effect, *p* < 0.01) and from 60% (pre-training) to 27% (T1) and 0% (T5) for the HFHI group (trial effect, *p* < 0.001). Planned comparisons demonstrated significant fall reduction between pre-training and T1 trial for the LFLI group (*X*^2^ = 4.16, *p* < 0.05), however, no significant difference was noted in the HFHI group (*X*^2^ = 2.28, *p* > 0.05). Both groups demonstrated a reduction in falls between T1 and T5 trials, however, this was not significant for either group (HFHI: *X*^2^ = 2.25, *p* > 0.05 and LFLI: *X*^2^ = 2.25, *p* > 0.05). Falls reduced significantly between the pre-training and T5 trials for both HFHI (*X*^2^ = 7.11, *p* < 0.05) and LFLI groups (*X*^2^ = 8.1, *p* < 0.05). There was a between group difference in falls on the pre-training trial (*X*^2^ = 5.72, *p* < 0.05), but no difference was observed at T1 (*X*^2^ = 0.99, *p* > 0.05) and T5 trials (*X*^2^ = 1.41, *p* > 0.05).

The mixed model analysis for number of compensatory steps demonstrated a significant trial effect (*p* < 0.01) during the early phase, however, there was no group effect (*p* > 0.05) or group*trial interaction (*p* > 0.05) (Fig. 2b). Within group analysis revealed a significant reduction in compensatory steps from pre-training trial to T5 for both HFLI and LFLI groups (trial effect, *p* < 0.0001 for HFHI; *p* < 0.05 for LFLI). Planned comparisons indicated a significant reduction in the number of compensatory steps for the HFHI group between pre-training and T5 trials (*p* < 0.001) and between T1 and T5 trials (*p* < 0.05), however, no difference was noted between pre-training and T1 trials (*p* > 0.05). While the LFLI group demonstrated significant reduction in compensatory steps between T1 and T5 trials (*p* < 0.05), no difference was noted between pre-training and T1 trials (*p* > 0.05) There was no between group difference in the number of steps at pre-training, T1, and T5 trials (*p* > 0.05).

**Fig. 3:**
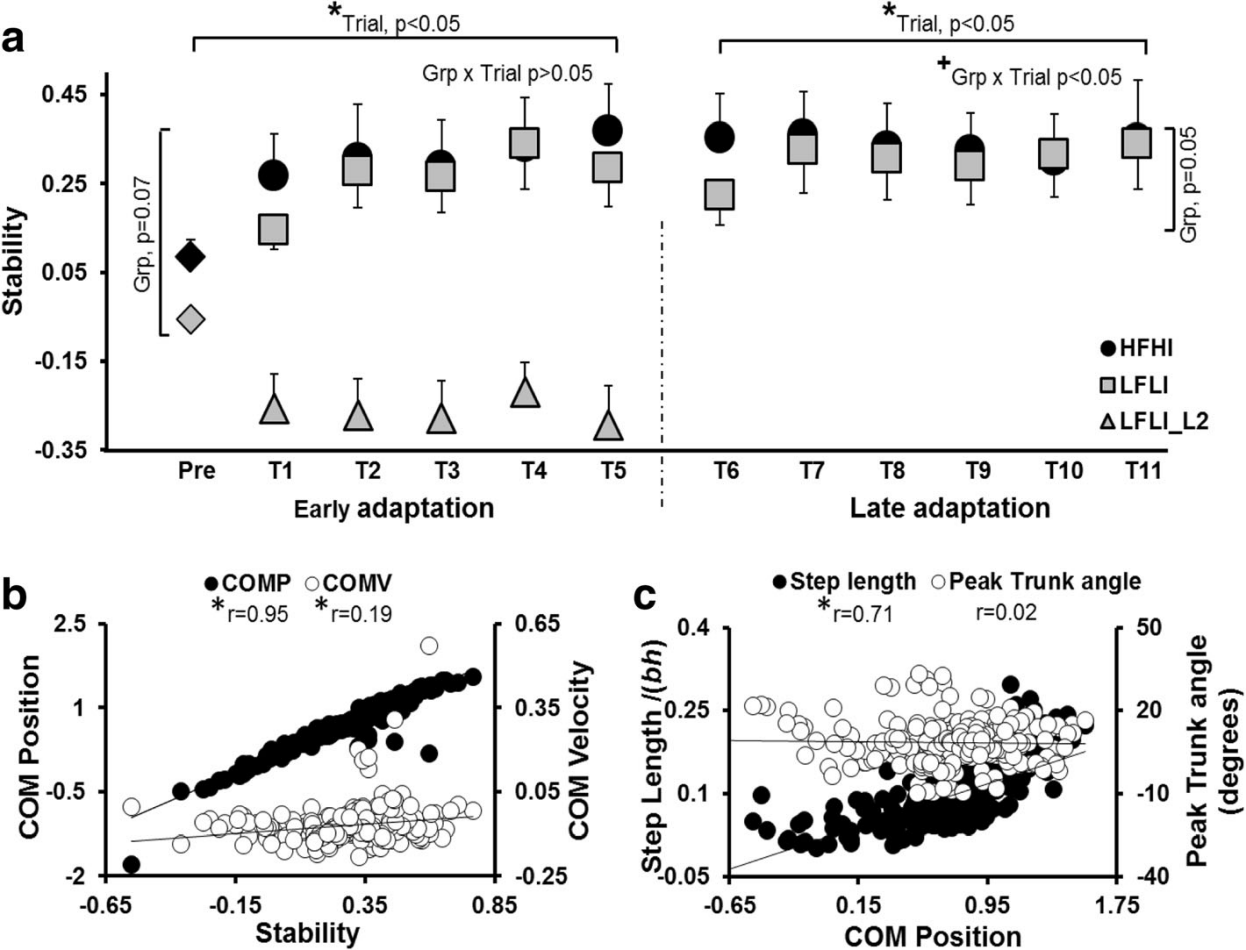
**a** Demonstrates the change in stability at touchdown (TD) during early adaptation (from pre-training to T5) and late adaptation (T6 and T11) for both groups. **b** Shows the relationship between COM position and velocity at TD relative to BOS with stability at TD, during T1-T11 training trials for both groups together. **c** Shows the relationship between step length and trunk angle with COM position at TD for both groups together. For 3a, * indicates a significant main effect of trial, and + indicates significant group x trial interaction. For 3b and c, *indicates significant correlation between variables with *p*<0.05

The mixed model for stability analysis demonstrated a significant trial effect (*p* < 0.0001) and near significant group effect (*p* = 0.07) for the early phase (Fig. 3a) with no group*trial interaction (*p* > 0.05). Within group analysis determined a significant trial effect for both the HFHI and the LFLI groups with a significant improvement in stability from pre-training to T5 trial (*p* < 0.0001). Further planned comparisons demonstrated a significant increase in stability from pre-training to T1 (*p* < 0.05), from T1 to T5 (*p* < 0.05), and from pre-training to T5 trial (*p* < 0.001) for the HFHI group. The LFLI group demonstrated a significant increase in stability between pre-training and T5 trial (*p* < 0.05), with no difference between pre-training and T1 (*p* > 0.05) or between T1 and T5 trials (*p* > 0.05). At the pre-training trial, stability was higher for the HFHI than the LFLI group (*p* = 0.07) with no between group difference at T1 (*p* > 0.05) and T5 trials (*p* > 0.05).

#### Late adaptation

The GEE model demonstrated no significant trial effect (*p* > 0.05) for fall incidence during the late phase (from T6 to T11 trials), however a significant group effect (*p* < 0.0001) and group*trial interaction (*p* < 0.0001) was observed (Fig. 2a). There was no between group difference noted at T6 (*X*^2^ = 1.41, *p* > 0.05) and T11 trials (*X*^2^ = 1.41, *p* > 0.05) for the HFHI and the LFLI groups. The mixed model analysis for number of compensatory steps demonstrated no significant trial effect (*p* > 0.05), group effect (*p* > 0.05), or group*trial interaction (*p* > 0.05) during the late adaptation phase (Fig. 2b).

For stability a significant trial effect (*p* < 0.05) and group*trial interaction (*p* < 0.05), with a near significant group effect (*p* = 0.05) was seen (Fig. 3a). Within group analysis demonstrated significant trial effect for the LFLI group (*p* < 0.05), demonstrating improvement in stability from T6 to T11, however not for the HFHI group (*p* > 0.05). Planned comparison revealed a significant increase in stability for the LFLI group during the late phase (*p* < 0.05).

#### Mechanism of adaptation in stability

A significant correlation was exhibited between COM position and stability (r = 0.95, *p* < 0.001) as well as between COM velocity and stability (r = 0.19, *p* < 0.001) at TD (Fig. 3b). There was a significant correlation between step length and COM position such that a longer step length resulted in an increase in COM position (r = 0.71, *p* < 0.05) (Fig. 3c). However, there was no correlation observed between COM position and trunk angle (r = 0.02, *p* > 0.05) (Fig. 3c).

### Scaling and retention

**Fig. 4:**
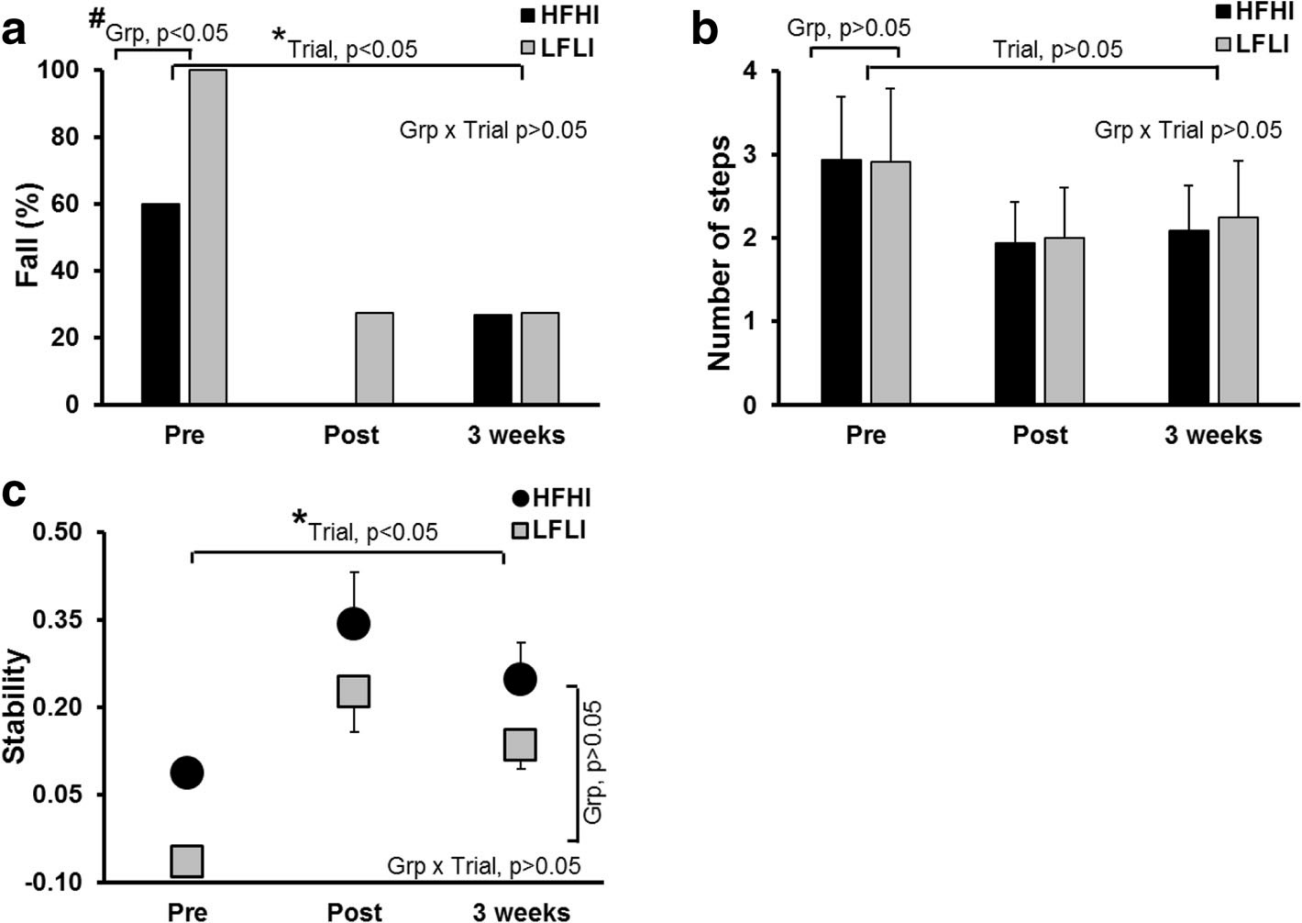
Scaling and retention. **a** Indicates the change in fall percentage from pre-training to post-training and after 3 weeks of retest trials. Both groups demonstrated a significant reduction in the fall outcome from pre-training to retest trials. **b** Demonstrates the change in the number of compensatory steps taken. **c** Demonstrates the change in stability at touchdown. There was a significant scaling and retention effect noted in the stability of both the HFHI and LFLI groups. *, indicates a significant main effect of trial, and # indicates a significant main effect of group

The GEE model demonstrated a significant group effect (*p* < 0.01), trial effect (*p* < 0.01) with no significant group*trial interaction (*p* > 0.05) for incidence of falls between pre-training, post-training, and retest trials (Fig. 4a). In both groups, there was a significant reduction in falls from pre-training to post-training trials for the HFHI group (*X*^2^ = 7.11, *p* < 0.05, 60 to 0%) and LFLI group (*X*^2^ = 6.12, *p* < 0.05, 100 to 27%). There was an increase in falls for the HFHI group from post-training to retest trials (*X*^2^ = 2.25, *p* = 0.06, 0 to 27%), however there was no difference in falls for the LFLI group (*X*^2^ = 0, *p* > 0.05). The HFHI group demonstrated fall reduction from pre-training (60%) to retest trials (27%) (*X*^2^ = 2.28, p = 0.06). Similarly, the LFLI group demonstrated fall reduction from pre-training to retest trials (*X*^2^ = 2.25, p = 0.06). Between the HFHI and LFLI groups, there was a significant difference in falls for the pre-training trial (*X*^2^ = 5.72, *p* < 0.05), but there was no difference observed for post-training (*X*^2^ = 4.62, *p* > 0.05) and retest trials (*X*^2^ = 0.076, *p* > 0.05).

In addition to fall outcomes, the mixed model analysis for the number of compensatory steps also demonstrated no significant trial effect (*p* > 0.05), group effect (*p* > 0.05), or group*trial interaction (*p* > 0.05) for pre-training, post-training, and retest trials (Fig. 4b).

For stability, a significant trial effect (*p* < 0.05) with no group effect (*p* > 0.05) or group*trial interaction (*p* > 0.05) was seen (Fig. 4c). There was a significant increase in stability for the HFHI group from pre-training to post-training (*p* < 0.001) and from pre-training to retest trials (*p* < 0.05), however there was no difference in stability between the post-training and retest trials (*p* > 0.05). The LFLI group demonstrated a significant increase in stability from pre-training to post-training (*p* < 0.05) and a significant increase in stability from pre-training to retest trials (*p* < 0.06), but there was no difference between post-training and retest trials (*p* > 0.05).

## Discussion

The results of the study indicated that PwCS were able to demonstrate reactive adaptation to slip-like perturbations at different perturbation intensities depending on their level of motor impairment. The HFHI group demonstrated adaptation at a higher intensity whereas the LFLI group demonstrated similar adaptive gains in stability after adjusting (lowering) the perturbation intensity. Both groups significantly scaled their adaptive recovery responses to higher perturbation intensity immediately after training. In addition, they demonstrated retention of the learned motor behavior three weeks after training.

### Response to first slip-like perturbation

Chronic stroke survivors have demonstrated increased risk of falls due to inefficient recovery strategies such as reduced postural stability, poor vertical limb support, inefficient compensatory stepping, and increased dependence on external support when subjected to an unannounced external perturbation [18, 45]. In line with these findings, all participants in this study experienced a backward balance loss with a multiple stepping response on their first novel, pre-training slip, with 100% of participants in the LFLI group and 60% in the HFHI group experiencing a fall (Fig. 2a, b). Such high fall incidence could be explained by the inability of the stroke survivors to control their COM state stability through compensatory stepping and/or trunk kinematics, resulting in a more posterior COM position and velocity relative to BOS at touchdown (see Fig. 3).

More importantly, fall incidence on the initial exposure to a high intensity, sudden slip-like perturbation was associated with the severity of motor impairment. The group with greater neuromuscular impairment, as indicated by lower CMSA scores [Mean(SD): 4(0.96)], showed relatively poor reactive balance control and, therefore, a higher fall incidence compared with the group with higher CSMA scores [Mean(SD): 4.93(1.12)]. Motor impairment after stroke is predominantly quantified by the ability to perform voluntary movements through complete or functional ROM with and without synergy. Higher motor impairment would indicate slower movements and/or reduced coordination of movements due to synergy. Indeed, researchers have shown that higher impairment levels are associated with longer movement completion times and greater movement errors [46–48]. Several studies have shown that scores on functional measures of upper extremities, lower extremities, and balance are related to the severity of motor impairment [49–53]. Thus, greater motor impairments could influence the ability to accurately position limbs or perform a specific movement within a given time constraint, which are both an integral part of balance recovery from sudden large perturbations.

### Adaptation across trials

Previous studies have reported an increased risk of falls in PwCS due to inefficient reactive balance control [18, 45]. Similarly, in the current study a significant proportion of PwCS experienced falls on their first novel, pre-training slip.

After the pre-training novel slip (16.75m/s^2^), the HFHI group demonstrated rapid adaptation to next lower perturbation intensity (12 m/s^2^) in the early phase (trial 1 to 5) (Figs. 2a & 3a), indicating adaptive improvement in reactive stability with decreased number of steps and reduced fall incidence. On the contrary, the LFLI group was unable to adapt to such high intensity (12 m/s^2^) and experienced a fall on every trial. Thus, the differences in performance level at a high (12 m/s^2^) perturbation intensity was affected by the severity of motor impairments; unlike LFLI group, preservation of neuromuscular control in the HFHI group could have facilitated rapid adaptation to a higher perturbation intensity in this group. However, when the perturbation intensity for the LFLI group was lowered one more level (7 .5m/s^2^), a similar trend of trial to trial adaptation was noted in both the groups. Note that even though the low functioning group was trained at a lower intensity, their stability was still less than the stability of the high functioning group on T1, and their stability remained lower on T5 as well. The LFLI group however, with repeated perturbations, continued to adapt in the late phase with a greater change in stability from T6-T11 than the HFHI group which seemed to reach a plateau in adaptation. (Fig. 3a).

Adaptive improvements in stability for both groups were associated with training-induced modulation of COM position and velocity (Fig. 3b) with COM position demonstrating a much greater correlation (r = 0.95) than velocity (r = 0.19). Such alteration of the COM position was observed to be regulated predominantly by change in the step length rather than change in trunk angle, suggesting that modulating step length was the preferred mode of improving their COM position in PwCS (Fig. 3c). The increase in step length throughout trials could have served a twofold function, repositioning the posteriorly shifted COM within the BOS and/or providing a larger moment arm for deceleration of the backward moving COM [11]. Unlike previously demonstrated in healthy young adults, PwCS exhibited minimal to no contribution from trunk angle for modulating the anterior shift in COM position with respect to the BOS [54, 55]. Our findings concur with previous perturbation training studies in healthy young and older adults, wherein similar contributions for modulation in the COM position were observed [54, 56].

### Effect of motor impairment on adaptation

Evidence suggests that severity of clinical impairment and functional recovery are related to the size and location of lesion which in turn is known to negatively influence overall motor performance [49, 57–63]. Based on this evidence, it can be postulated that severe motor impairment in the LFLI group may have resulted from larger residual lesions in the frontal cortex or damage to cerebellar-thalamic-cortical pathways resulting in failure to adapt at a higher perturbation intensity [64]. Previous evidence from locomotor studies suggests that large motor errors produced by high intensity training result in better outcomes than errors produced by low intensity training [65–69]. However, another line of evidence suggests that large motor errors, rather than facilitating learning, might actually impede learning [70]. Such impedance to learning on a higher perturbation intensity (12 m/s^2^), as seen in the LFLI group, may be related to more severe sensorimotor impairments. Relatively higher level of motor impairments may lead to failure in detecting motor mismatch or else limited ability to effectively modulate motor output in accordance with the sensory input (i.e. magnitude of motor error), resulting in the inability to adapt at a higher intensity.

The current results demonstrate that even PwCS with relatively severe motor impairments, have the potential to acquire reactive adaptation upon exposure to a suitable perturbation intensity. The LFLI group exhibited improved performance upon reducing the environmental threat and continued to demonstrate late adaptation with stability gains nearing those of the HFHI group at T11 trial. Our findings concur with previous findings demonstrating rapid training induced adaptive gains in gait speed in moderately impaired PwCS for the initial 24 sessions of locomotor training, followed by smaller adaptive gains during the later phase from the 24th to 36th session [71]. In comparison, severely impaired PwCS showed slow and constant improvement in gait speed until the last, 36th session. Thus, our findings, along with concurring previous studies, suggest the need for additional practice for acquisition of the motor task in the presence of greater motor impairment in order to accommodate for slower adaptation.

### Scaling and retention of adaptive responses

PwCS have demonstrated failure to modulate reactive responses upon exposure to novel, increasing perturbation intensities compared with age-matched healthy older adults [20]. However, we found that reactive balance training could enhance the ability to modulate recovery response to a higher perturbation intensity. There was a significant improvement in fall percentage and reactive stability for both groups when exposed to a higher level of perturbation immediately post-training. This indicates the intact ability of PwCS to scale their reactive adaptation based on perturbation intensity, even after a single session of training, and this is similar to results previously demonstrated in young adults [54] (Fig. 4).

The adaptation and scaling effects acquired from a single session of perturbation training were retained, with participants demonstrating reduced falls and improved stability control at their 3-week retest compared with their pre-training novel slip. Additionally, their ability to show scaling was maintained, as there was no difference between their post-perturbation slip (12 m/s^2^) and their retest slip (16.75/m^2^). A similar, but more robust, long term retention of acquired fall-resisting skills for up to 12 months was previously shown in healthy older adults following a single session of repeated over ground slip perturbations [23, 72].

Retention demonstrated in these previously published locomotor-perturbation studies allowed for modulation of both proactive and reactive responses [23, 72]. In such cases, it is proposed that the adaptation process is associated with a recalibration of the internal model of one’s stability limits and involves a shift from reliance on short- and long-loop reflex pathways within the spinal cord and brainstem to increased subcortical and cortical influences [73]. These newly acquired or enhanced sensorimotor relationships involving higher control systems, conscious control, and feed forward mechanisms can easily be consolidated and stored, resulting in longer-term retention [74, 75]. In healthy adults, the ability to acquire perturbation-induced reactive adaptations, through the use of a paradigm similar to ours has already been established [54, 76], however, the neurophysiological mechanisms underlying adaptation and retention purely within the reactive balance control system are less studied and are not well understood. [54, 73, 77].

### Mechanism of reactive adaptation and retention

Recent studies have established the importance of cortical modulation for regulating reactive responses [78–80]. It is postulated that for adaptation within a purely reactive system the process of updating the internal representation of stability limits for each repeated exposure would still be applicable resulting in formation of a motor repertoire that could be triggered when a similar sensory stimulus is received [81, 82]. Therefore, in our study, it is postulated that, after initial exposure to slip-like perturbations, the CNS extracts information related to the perturbation characteristics (acceleration, belt displacement, and direction) as well as information regarding the amount of lower limb and trunk movement required to achieve optimal post-slip stability [83, 84]. On subsequent exposure to familiar postural disturbances, the previous motor memory is recalled. Information regarding the magnitude of threat, coupled with the prediction of balance loss, helps in the selection of an appropriate recovery response and the subsequent modulation of body kinematics to resist the COM displacement [42]. It can be postulated that contextual familiarity such as the experiment instrument, laboratory settings, previous exposure to laboratory slip-like perturbations, similar sensorimotor experience from real life, and the use of the same perturbation direction for training and testing may have helped in scaling the reactive responses to a higher intensity of perturbation [54, 85–87].

Although the cerebral cortex is involved in feedback control of movements during reactive balance responses [78–80], our findings suggest that the CNS possesses the capacity to undergo changes to acquire reactive balance skills even after unilateral cerebral cortex injury. For those recovering from stroke, adapting to slip-like perturbations in some ways involves re-acquiring a skilled movement in the presence of a new configuration of neural networks. The exact neural substrates involved in re-acquisition of reactive balance skills are however not known. A recent functional magnetic resonance imaging study demonstrated evidence of activation changes within the parietal, prefrontal areas, cingulate gyri, cerebral cortices and cerebellar regions in healthy young adults post slip-perturbation training [88]. Unlike in healthy adults, the reorganization of neural networks while learning and the retention of new skilled movements after stroke occurs with compensatory increased activation of the contralateral brain areas [89]. Such changes in activation are predominantly observed in the M1 motor area [89], premotor cortex [90], and prefrontal areas [91]. While evidence of neural re-organization with motor adaptation after stroke is predominantly from an upper extremity visuomotor task learning [92], adaptation to novel balance tasks may involve similar processes. Our findings indicate that the potential of retaining single-session-induced reactive adaptations for at least several weeks is preserved even in chronic stroke survivors. Previous studies in healthy older adults have shown that although a single session of real-life-like slip perturbations induces longer-term changes, there is decay in motor memory over time with more robust retention (in the form of fall reduction) demonstrated for up to 6 months followed by a gradual increase in fall incidence at the retest session after 12 months [23, 72]. However, the impact of dosage (number of sessions and trials per session) on motor memory decay in individuals with acquired cortical lesion remains to be determined.

## Clinical application

Chronic stroke survivors have a greatly increased risk of falls, and therefore, a perturbation training paradigm to improve reactive balance has significant clinical importance. The operator-driven instrumented treadmill training during stance was designed to minimize proactive adaptations, thus enforcing reactive adaptations. Our findings indicate the preserved potential of re-training reactive balance to large external perturbations through a single training session in PwCS. Despite the baseline differences in reactive stability across impairment levels, even the severely impaired PwCS exhibited preserved implicit-error-based feedback-learning, resulting in reactive adaptation which could be subsequently scaled and retained to respond to more challenging perturbations. Rehabilitation interventions could exploit this intact, implicit motor-learning ability to improve reactive balance control and reduce fall risk.

Our findings must be interpreted with the understanding that there are some limitations. Firstly, although participants were included based on the confirmation of a cortical stroke, the exact extent, area, and size of the lesion was unknown. Secondly, the pre-post, non-randomized study design without a control group (either receiving conventional or no training) warrants future randomized controlled studies to establish efficacy of perturbation training paradigm for fall-risk reduction in PwCS. Thirdly, this study provide preliminary evidence regarding acquisition and retention of reactive adaptation in PwCS. However, this study being first of its kind did not examine generalization to other perturbation directions and contexts in a laboratory setting or translation of real life falls. Given that PwCS can generalize acquired locomotor training to non-trained contexts [93, 94], it is possible that they could also generalize the perturbation-induced adaptation to other conditions, as previously seen in healthy adults [95–98]. PwCS may perhaps require additional training dosage to induce a similar extent of generalization. Future studies need to be conducted to determine the same.

## Conclusion

Our results suggest that chronic stroke survivors with both mild-moderate and severe lower limb impairments have the intact capacity to acquire and retain reactive adaptations to a similar extent in order to decrease their risk of falls. However, the more impaired PwCS, compared with the less impaired, might require a gradual increase in training intensity to induce significant reactive adaptations to large-magnitude stance perturbations.

## Abbreviations

BBS: Berg Balance Scale
BOS: Base of support
CMSA: Chedoke-McMaster Stroke Assessment scale
CNS: Central nervous system
COM: Center of mass
HFHI: High functioning, high intensity group
LFLI: Low functioning, low intensity group
PwCS: People with chronic stroke
TD: Touchdown
TUG: Timed up and go test
